# Supplementary material for: Upregulation of MMP3 Promotes Cisplatin Resistance in Ovarian Cancer
Source: Int J Mol Sci. 2025 Apr 24;26(9):4012. doi: 10.3390/ijms26094012 (PMC12071843; doi:10.3390/ijms26094012)
Supplement: Supplementary file 1 [file ijms-26-04012-s001.zip › ijms-3495604-Supplementary.pdf]

Table S1A. Top 50 differentially expressed genes (DEGs) associated with the siRNA-mediated MMP3 knockdown in OVCAR3CIS cells, ranked by adjusted p-value (p-adj).

Gene name	ID	log2FoldChange	p-adj
SCAMP5	ENSG00000198794	1.060988	1.76×10^{-51}
NEFH	ENSG00000100285	1.139433	2.61×10^{-40}
BTG2	ENSG00000159388	-1.09713	4.09×10^{-26}
VASP	ENSG00000125753	-1.05933	5.52×10^{-25}
ITGA5	ENSG00000161638	-1.00827	5.68×10^{-24}
WWTR1	ENSG00000018408	-1.03085	2.2×10^{-22}
PHF21B	ENSG00000056487	1.042219	9.14×10^{-22}
RPS6KA4	ENSG00000162302	-1.02319	1.39×10^{-21}
DLX2	ENSG00000115844	-1.22783	1.52×10^{-20}
RELL2	ENSG00000164620	1.035967	9.42×10^{-16}
KCNQ2	ENSG00000075043	1.043291	3.80×10^{-15}
SERPINB9	ENSG00000170542	-1.02407	1.95×10^{-11}
SNCB	ENSG00000074317	1.281406	5.91×10^{-11}
SVOP	ENSG00000166111	4.904506	4.58×10^{-10}
MPC1	ENSG00000060762	1.037979	6.96×10^{-10}
GPR3	ENSG00000181773	-1.12902	7.66×10^{-10}
ERO1A	ENSG00000197930	-1.01424	8.17×10^{-10}
C3orf14	ENSG00000114405	1.073282	9.45×10^{-10}
LMLN	ENSG00000185621	-1.0919	2.97×10^{-9}
FGF7P3	ENSG00000204837	1.163488	9.88×10^{-9}
IGF2R	ENSG00000197081	-1.005	1.47×10^{-8}
GNG7	ENSG00000176533	1.066952	1.50×10^{-8}
DDI2	ENSG00000197312	-1.07772	2.77×10^{-8}
AL596244.1	ENSG00000261534	2.73641	4.96×10^{-8}
CPLX2	ENSG00000145920	4.371552	6.44×10^{-8}
GALNT6	ENSG00000139629	-1.04708	6.77×10^{-8}
EPN3	ENSG00000049283	1.123567	1.65×10^{-7}
PACRG	ENSG00000112530	1.249935	2.68×10^{-7}
CD82	ENSG00000085117	1.024103	2.80×10^{-7}
RTBDN	ENSG00000132026	1.151173	2.92×10^{-7}
C10orf91	ENSG00000180066	1.195648	5.12×10^{-7}
GNG2	ENSG00000186469	1.478103	1.05×10^{-6}
FSD2	ENSG00000186628	2.032841	1.26×10^{-5}
PTPRN2	ENSG00000155093	3.651893	1.68×10^{-5}
LINC01003	ENSG00000261455	1.036396	2.04×10^{-5}
TMEM2	ENSG00000135048	-1.09805	2.31×10^{-5}
HGC6.3	ENSG00000235994	2.110223	2.97×10^{-5}
DDIT4L	ENSG00000145358	1.327822	3.25×10^{-5}
IL3RA	ENSG00000185291	1.079714	3.43×10^{-5}
CNIH3	ENSG00000143786	1.046245	3.91×10^{-5}

RIPPLY2	ENSG00000203877	1.00697	6.57×10^{-5}
F2RL1	ENSG00000164251	-1.20831	6.73×10^{-5}
ANKRD24	ENSG00000089847	1.159805	7.54×10^{-5}
SLC26A10	ENSG00000135502	-1.04869	1.17×10^{-4}
AC145207.5	ENSG00000263731	-1.03677	1.35×10^{-4}
AC137723.1	ENSG00000264569	2.727824	1.43×10^{-4}
SMTNL2	ENSG00000188176	2.663185	1.73×10^{-4}
PITX3	ENSG00000107859	1.338765	2.22×10^{-4}
WEE2-AS1	ENSG00000228775	1.170604	2.28×10^{-4}
ABLIM2	ENSG00000163995	1.000565	4.34×10^{-4}

The total number of DEG was 144, identified exclusively in the NC vs siMMP3 comparisons.

Table S1B. Top 50 DEGs associated with MMP3-dependent cisplatin response in OVCAR3CIS cells, ranked by adjusted p-value (p-adj).

Gene name	ID	log2FoldChange	p-adj
CHGB	ENSG00000089199	3.603976	9.84×10^{-154}
CHRNB2	ENSG00000160716	4.26102	7.07×10^{-137}
SCG3	ENSG00000104112	5.04094	2.42×10^{-130}
AP3B2	ENSG00000103723	2.221219	4.52×10^{-122}
SYP	ENSG00000102003	2.309564	1.06×10^{-121}
STMN3	ENSG00000197457	2.493578	1.14×10^{-110}
RUNDC3A	ENSG00000108309	3.001584	2.17×10^{-102}
ACTL6B	ENSG00000077080	4.645848	1.06×10^{-96}
XKR7	ENSG00000260903	4.393692	3.97×10^{-85}
MAPK8IP2	ENSG00000008735	2.266975	1.26×10^{-72}
CPLX1	ENSG00000168993	3.580086	2.59×10^{-71}
NFE2L2	ENSG00000116044	-1.23975	2.19×10^{-59}
UNC79	ENSG00000133958	2.025565	1.71×10^{-57}
MAPK8IP1	ENSG00000121653	1.378141	3.56×10^{-55}
PAX5	ENSG00000196092	3.248805	3.04×10^{-52}
CHGA	ENSG00000100604	3.290491	2.07×10^{-49}
AC005696.4	ENSG00000277200	3.286964	1.39×10^{-48}
FAM57B	ENSG00000149926	2.656496	3.01×10^{-48}
INIP	ENSG00000148153	-1.25455	9.29×10^{-47}
DPY19L1	ENSG00000173852	-1.36383	2.2×10^{-44}
PYGM	ENSG00000068976	2.760726	3.97×10^{-44}
TMEM145	ENSG00000167619	2.10895	1.96×10^{-42}
CBFB	ENSG00000067955	-1.20494	9.01×10^{-41}
SRRM3	ENSG00000177679	2.578725	9.11×10^{-41}
CTGF	ENSG00000118523	-1.88678	1.42×10^{-40}
KCNC1	ENSG00000129159	4.687167	3.99×10^{-39}
CD34	ENSG00000174059	2.370871	2.47×10^{-37}
REEP3	ENSG00000165476	-1.18799	8.03×10^{-36}
TMEM229B	ENSG00000198133	1.96527	2.4×10^{-35}

RFPL1S	ENSG00000225465	1.800858	4.25×10^{-33}
CNRIP1	ENSG00000119865	1.302044	4.62×10^{-33}
KIF25-AS1	ENSG00000229921	1.968988	5.31×10^{-33}
DISP2	ENSG00000140323	1.418821	1.06×10^{-32}
EOGT	ENSG00000163378	-1.35873	4.57×10^{-32}
MBTPS1	ENSG00000140943	-1.1555	2.51×10^{-31}
TMEM198	ENSG00000188760	1.960787	2.55×10^{-31}
GPD2	ENSG00000115159	-1.44264	7.61×10^{-31}
HHATL	ENSG00000010282	4.610086	1.06×10^{-30}
STYX	ENSG00000198252	-1.26939	1.09×10^{-30}
SGPL1	ENSG00000166224	-1.45354	1.94×10^{-30}
E2F5	ENSG00000133740	-1.00088	1.21×10^{-29}
FNDC5	ENSG00000160097	1.378102	3.29×10^{-29}
ITGB1	ENSG00000150093	-1.42047	4.91×10^{-29}
CARMIL3	ENSG00000186648	1.973566	9.04×10^{-29}
SEZ6	ENSG00000063015	3.790665	1.62×10^{-27}
LMOD1	ENSG00000163431	1.44903	2.57×10^{-27}
EXOC3L1	ENSG00000179044	2.209226	4.13×10^{-27}
GXYLT1	ENSG00000151233	-1.20971	4.81×10^{-27}
HCN2	ENSG00000099822	1.687241	9.00×10^{-27}
FIBIN	ENSG00000176971	1.161435	2.49×10^{-26}

The total number of DEGs were 262, identified as common in the NC+CIS vs siMMP3+CIS and NC vs siMMP3 comparisons.

Table S1C. Top 50 DEGs associated with cisplatin response in OVCAR3CIS cells, ranked by adjusted p-value (p-adj).

Gene name	ID	log2FoldChange	p-adj
CDC25A	ENSG00000164045	-1.0636	4.63×10^{-44}
ANKRD1	ENSG00000148677	-1.0090	3.96×10^{-39}
ACTR2	ENSG00000138071	-1.1531	3.37×10^{-37}
GFPT1	ENSG00000198380	-1.0276	1.58×10^{-34}
KCNC3	ENSG00000131398	1.1819	9.43×10^{-33}
CARNMT1	ENSG00000156017	-1.0228	6.55×10^{-32}
PLEKHB2	ENSG00000115762	-1.0282	2.73×10^{-31}
PODXL2	ENSG00000114631	1.0052	1.41×10^{-27}
LIMA1	ENSG00000050405	-1.0028	5.53×10^{-27}
REEP5	ENSG00000129625	-1.0534	3.17×10^{-26}
UEVLD	ENSG00000151116	-1.0062	8.64×10^{-25}
SBK1	ENSG00000188322	1.1215	9.09×10^{-22}
POLR3G	ENSG00000113356	-1.0585	7.21×10^{-21}
ADAMTSL4	ENSG00000143382	1.0051	7.96×10^{-19}
MOSPD3	ENSG00000106330	1.0839	3.99×10^{-18}
TRIP11	ENSG00000100815	-1.0167	3.20×10^{-17}
TIMM10	ENSG00000134809	-1.1039	1.03×10^{-16}
PDCD4	ENSG00000150593	-1.0309	1.07×10^{-16}

F11R	ENSG00000158769	-1.0167	1.46×10^{-16}
CNNM1	ENSG00000119946	1.0436	1.82×10^{-15}
HAPLN3	ENSG00000140511	1.0483	4.63×10^{-44}
TJP3	ENSG00000105289	1.0044	3.96×10^{-39}
KCNN1	ENSG00000105642	1.0605	3.37×10^{-37}
ADAMTS10	ENSG00000142303	1.1098	1.58×10^{-34}
PC	ENSG00000173599	1.0161	9.43×10^{-33}
DNM1	ENSG00000106976	1.0587	6.55×10^{-32}
PCLO	ENSG00000186472	1.0116	2.73×10^{-31}
GNG4	ENSG00000168243	1.0048	1.41×10^{-27}
SYN1	ENSG00000008056	1.0906	5.53×10^{-27}
AHNAK2	ENSG00000185567	1.0480	3.17×10^{-26}
DDIT3	ENSG00000175197	1.1036	8.64×10^{-25}
FAM46B	ENSG00000158246	1.2420	9.09×10^{-22}
HIST3H2A	ENSG00000181218	1.0533	7.21×10^{-21}
C11orf87	ENSG00000185742	1.1889	7.96×10^{-19}
AC006538.1	ENSG00000261342	1.2599	3.99×10^{-18}
MLXIPL	ENSG00000009950	1.2004	3.20×10^{-17}
AP001005.3	ENSG00000282965	1.6065	1.03×10^{-16}
GPRC5C	ENSG00000170412	1.0113	1.07×10^{-16}
CTH	ENSG00000116761	1.1373	1.46×10^{-16}
AZIN2	ENSG00000142920	1.4236	1.82×10^{-15}
PSTPIP2	ENSG00000152229	1.0265	4.63×10^{-44}
FCGBP	ENSG00000275395	1.4488	3.96×10^{-39}
VAX2	ENSG00000116035	1.0287	3.37×10^{-37}
PLIN4	ENSG00000167676	1.3814	1.58×10^{-34}
CDKN1C	ENSG00000129757	1.0948	9.43×10^{-33}
TMEM63C	ENSG00000165548	1.1317	6.55×10^{-32}
GOLT1A	ENSG00000174567	1.0705	2.73×10^{-31}
ACHE	ENSG00000087085	1.0991	1.41×10^{-27}
PPP1R1A	ENSG00000135447	1.4262	5.53×10^{-27}
ITGA10	ENSG00000143127	1.3774	3.17×10^{-26}

The total number of DEGs was 171, identified exclusively in the NC+CIS vs siMMP3+CIS comparisons.

Table S2. Top 50 differentially expressed genes associated to cisplatin treatment in OVCAR3CIS cells, ranked by adjusted p-value (padj).

Gene name	ID	log2FoldChange	padj
PIF1	ENSG00000140451	-1.07388	2.78×10^{-14}
PARD3B	ENSG00000116117	-1.97174	2.90×10^{-14}
MACROD2	ENSG00000172264	-1.1616	9.57×10^{-14}
PQLC1	ENSG00000122490	1.159497	8.35×10^{-13}
ANTXR1	ENSG00000169604	-1.12542	4.18×10^{-12}
BBS9	ENSG00000122507	-1.3223	9.80×10^{-12}
PDE11A	ENSG00000128655	-2.03624	2.32×10^{-11}

HES6	ENSG00000144485	1.231927	2.54×10^{-11}
CNN1	ENSG00000130176	1.356784	5.79×10^{-11}
SNX29	ENSG00000048471	-1.32565	8.67×10^{-11}
ABAT	ENSG00000183044	-1.53827	1.68×10^{-10}
BTBD9	ENSG00000183826	-1.09926	2.04×10^{-10}
HHAT	ENSG00000054392	-1.47757	3.45×10^{-10}
RIPPLY3	ENSG00000183145	1.074943	5.45×10^{-10}
MAML3	ENSG00000196782	-1.45673	3.14×10^{-9}
LTBP2	ENSG00000119681	-1.57506	6.95×10^{-9}
FGGY	ENSG00000172456	-1.05718	8.97×10^{-9}
NAALADL2	ENSG00000177694	-1.56056	1.69×10^{-8}
NEK11	ENSG00000114670	-1.02193	1.88×10^{-8}
XAF1	ENSG00000132530	-3.72662	1.88×10^{-8}
ALPK1	ENSG00000073331	-1.044	1.51×10^{-7}
CPQ	ENSG00000104324	-1.13952	2.96×10^{-7}
PYM1	ENSG00000170473	1.041945	6.26×10^{-7}
ABCA1	ENSG00000165029	-1.35264	1.54×10^{-6}
SSPO	ENSG00000197558	-1.45529	8.27×10^{-6}
TCP11L2	ENSG00000166046	-1.8505	1.01×10^{-5}
COL4A6	ENSG00000197565	-1.38145	1.45×10^{-5}
MYO15B	ENSG00000266714	-1.49682	1.79×10^{-5}
PCDHGA10	ENSG00000253846	-1.2602	1.88×10^{-5}
NRXN1	ENSG00000179915	-1.05891	2.62×10^{-5}
ANK2	ENSG00000145362	-1.30297	4.60×10^{-5}
C5AR1	ENSG00000197405	-1.01945	4.72×10^{-5}
AL049840.4	ENSG00000269958	1.054955	5.16×10^{-5}
ACBD7	ENSG00000176244	-1.03044	6.76×10^{-5}
SCN3A	ENSG00000153253	-1.41385	7.13×10^{-5}
RF02250	ENSG00000276048	-1.57541	7.58×10^{-5}
AC139887.2	ENSG00000249592	-1.43771	1.23×10^{-4}
FBXL20	ENSG00000108306	-1.13109	1.52×10^{-4}
SERPINB8	ENSG00000166401	1.016002	1.56×10^{-4}
UST	ENSG00000111962	-1.42516	1.74×10^{-4}
AL357033.4	ENSG00000277496	-1.96181	2.1×10^{-4}
AP1M2	ENSG00000129354	1.009173	2.54×10^{-4}
CFAP47	ENSG00000165164	-1.07608	3.11×10^{-4}
COL7A1	ENSG00000114270	-1.12132	3.12×10^{-4}
FAM241A	ENSG00000174749	1.177978	3.62×10^{-4}
OCA2	ENSG00000104044	-1.26531	4.85×10^{-4}
DNAH1	ENSG00000114841	-1.19549	5.00×10^{-4}
MAGI2	ENSG00000187391	-1.30011	1.07×10^{-3}
CSMD3	ENSG00000164796	-1.07817	1.13×10^{-3}
SAMD9L	ENSG00000177409	-1.35636	1.24×10^{-3}

The total number of DEGs was 101, identified exclusively in the NC vs cis comparisons.

Table S3. Consistently DEGs across all comparisons, ranked by adjusted p-value (padj).

Gene name	ID	log2FoldChange	padj
AL445490.1	ENSG00000225886	-4.569781454	6.95×10^{-9}
NMNAT2	ENSG00000157064	-1.11954943	5.53×10^{-6}
ITGB8	ENSG00000105855	-1.027190179	2.33×10^{-5}
CEND1	ENSG00000184524	-2.137671086	2.69×10^{-5}

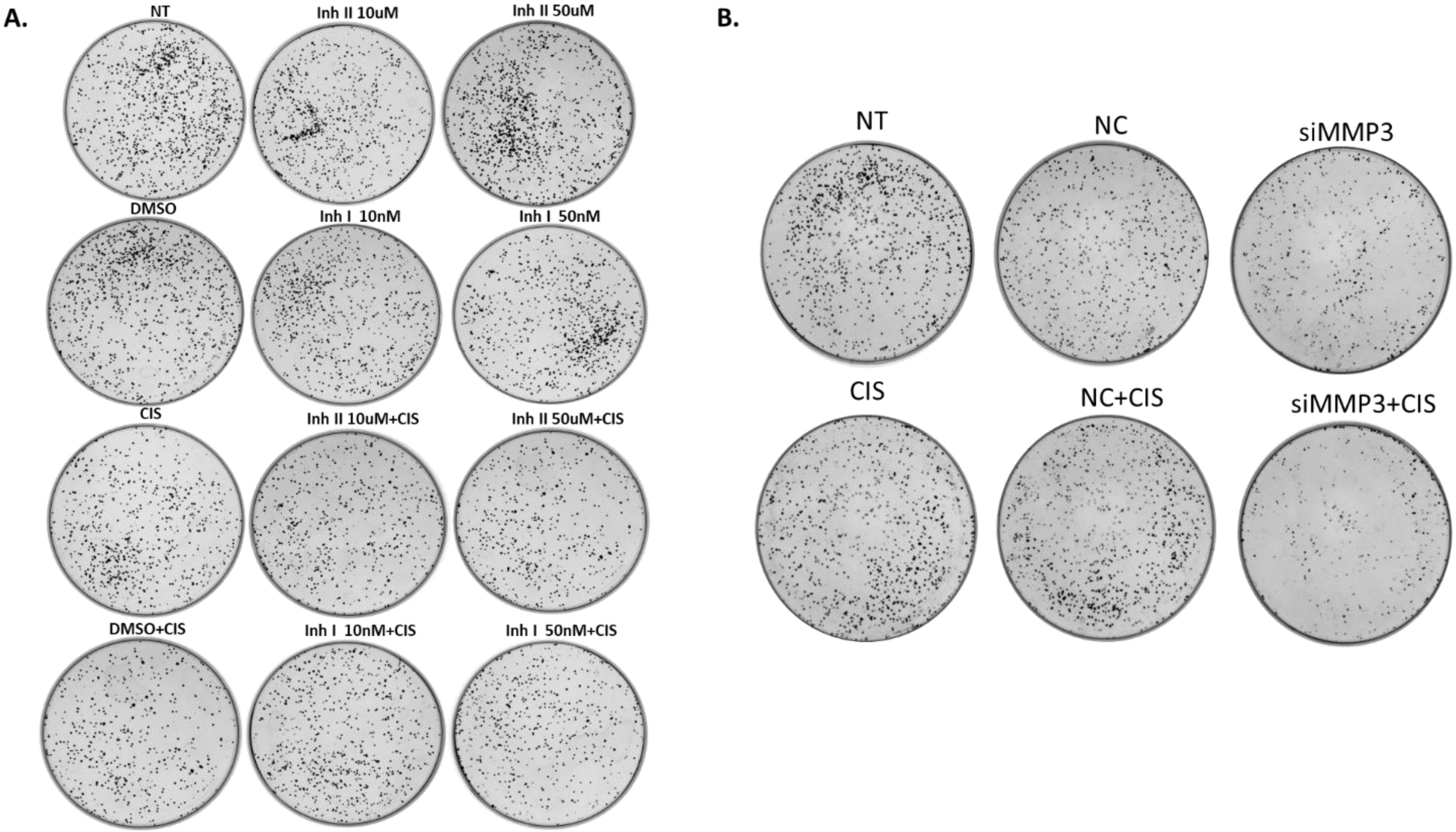

Figure S1. *Colony formation assay visualization following (A) MMP3 activity inhibition with small molecule inhibitors and (B) MMP3 siRNA transfection with and without cisplatin in OVCAR3CIS cells.*

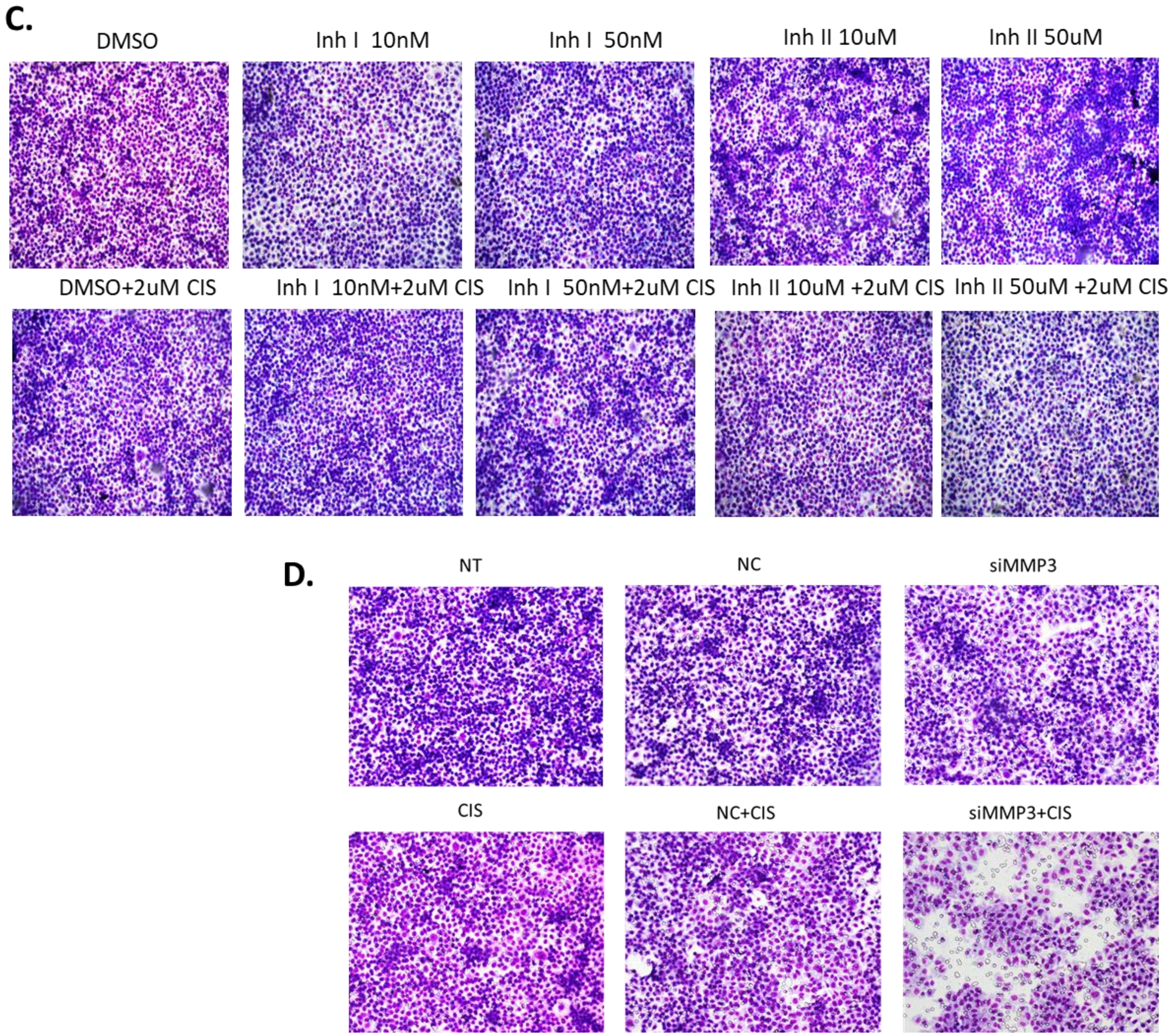

Figure S1. *Invasion ability assay visualization following (C) MMP3 activity inhibition with small molecule inhibitors and (D) MMP3 siRNA transfection with and without cisplatin in OVCAR3CIS cells.*

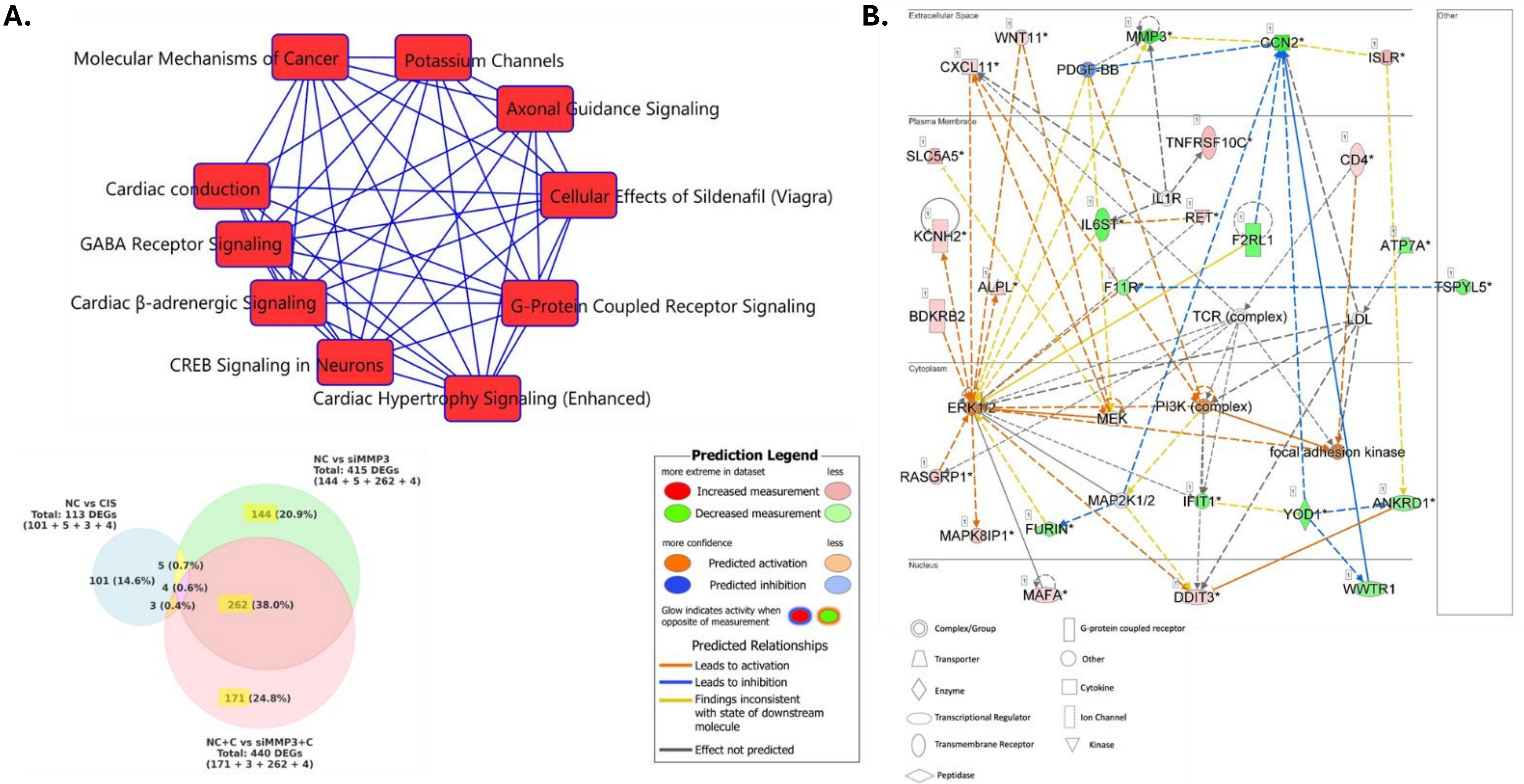

Figure S2. Downstream effectors following MMP3-siRNA knockdown and cisplatin-dependent protein associations of MMP3 in OVCAR3CIS cells. *(A) Top 10 canonical pathways identified by ingenuity pathway analysis of siRNA-mediated knockdown of MMP3 with and without cisplatin in OVCAR3CIS cells; (B) Top scored network (from 10 total networks) associated to MMP3 silencing in OVCAR3CIS cells with and without cisplatin. Relevant transcript are shown by subcellular location. Gene symbols with asterisk (*) correspond to the transcript identified in the RNA-seq. Indirect associations are represented by dashed lines while direct associations are represented by solid lines. Green icons: decreased; Red icons: increased; Blue icons: Predicted inhibition; Orange icons: Predicted activation. Highlighted in yellow within the Venn diagrams are the numbers of the DEGs subjected to IPA analysis.*

A.

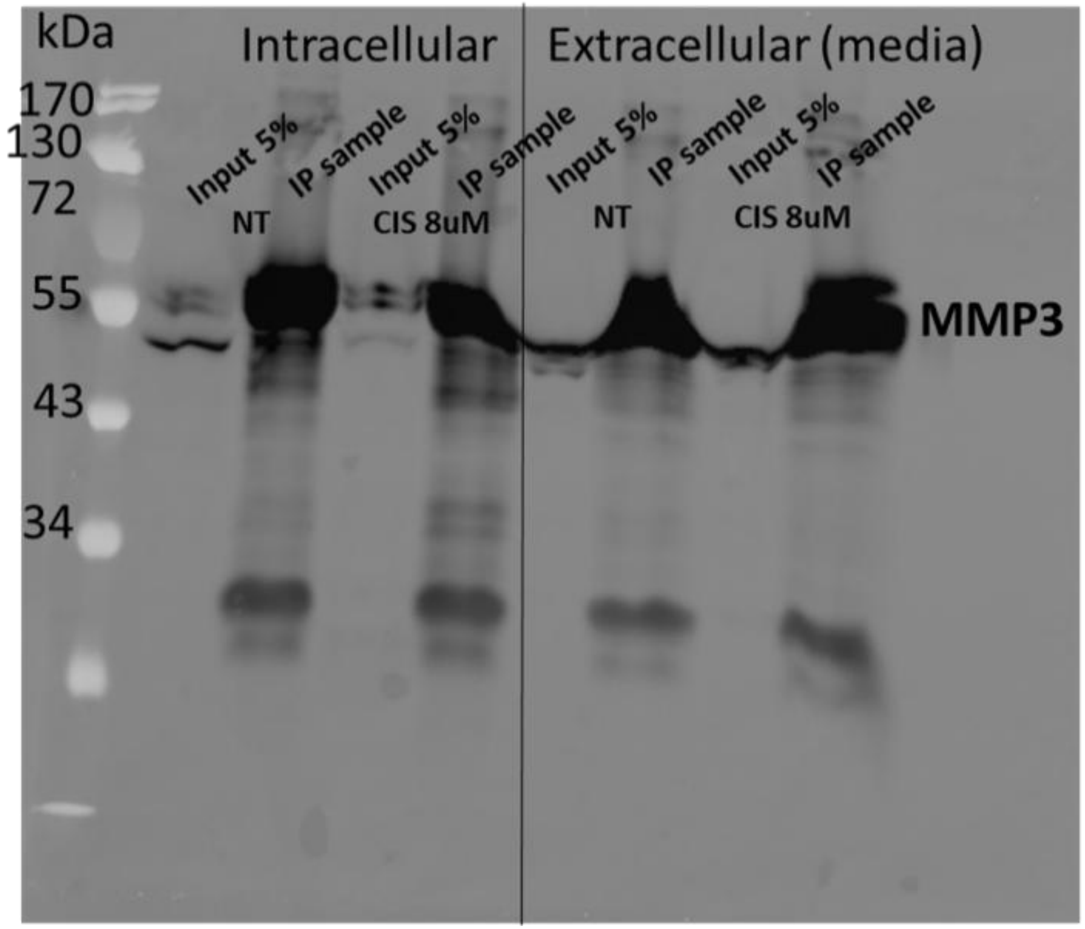

Figure S3. *Immunoprecipitation (IP) of intracellular and extracellular MMP3 validated by western blot analysis.*
